# Molecular characterisation of *Mycobacterium tuberculosis *isolates in the First National Survey of Anti-tuberculosis Drug Resistance from Venezuela

**DOI:** 10.1186/1471-2180-6-90

**Published:** 2006-10-10

**Authors:** Liselotte Aristimuño, Raimond Armengol, Alberto Cebollada, Mercedes España, Alexis Guilarte, Carmen Lafoz, María A Lezcano, María J Revillo, Carlos Martín, Carmen Ramírez, Nalin Rastogi, Janet Rojas, Albina Vázques de Salas, Christophe Sola, Sofía Samper

**Affiliations:** 1 Escuela de Medicina, Universidad Centroccidental Lisandro Alvarado, Venezuela; 2 Programa Nacional Integrado de Control de la Tuberculosis, MSDS, Venezuela; 3 Grupo de Genética de Micobacterias, Departamento de Microbiología, Medicina Preventiva y Salud Pública, Universidad de Zaragoza, España; 4 Servicio de Microbiología, Hospital Universitario Miguel Servet, Zaragoza, España; 5 Unité de la Tuberculose et des Mycobactéries, Institut Pasteur, Pointe-à-Pitre, Guadeloupe

## Abstract

**Background:**

Molecular typing of *Mycobacterium tuberculosis *strains has become a valuable tool in the epidemiology of tuberculosis (TB) by allowing detection of outbreaks, tracking of epidemics, identification of genotypes and transmission events among patients who would have remained undetected by conventional contact investigation. This is the first genetic biodiversity study of *M. tuberculosis *in Venezuela. Thus, we investigated the genetic patterns of strains isolated in the first survey of anti-tuberculosis drug-resistance realised as part of the Global Project of Anti-tuberculosis Drug Resistance Surveillance (WHO/IUATLD).

**Results:**

Clinical isolates (670/873) were genotyped by spoligotyping. The results were compared with the international spoligotyping database (SpolDB4). Multidrug resistant (MDR) strains (14/18) were also analysed by IS*6110*-RFLP assays, and resistance to isoniazid and rifampicin was characterised.

Spoligotyping grouped 82% (548/670) of the strains into 59 clusters. Twenty new spoligotypes (SITs) specific to Venezuela were identified. Eight new inter-regional clusters were created. The Beijing genotype was not found. The genetic network shows that the Latin American and Mediterranean family constitutes the backbone of the genetic TB population-structure in Venezuela, responsible of >60% of total TB cases studied. MDR was 0.5% in never treated patients and 13.5% in previously treated patients. Mutations in *rpoB *gene and *katG *genes were detected in 64% and 43% of the MDR strains, respectively.

Two clusters were found to be identical by the four different analysis methods, presumably representing cases of recent transmission of MDR tuberculosis.

**Conclusion:**

This study gives a first overview of the *M. tuberculosis *strains circulating in Venezuela during the first survey of anti-tuberculosis drug-resistance. It may aid in the creation of a national database that will be a valuable support for further studies.

## Background

The World Health Organisation (WHO) estimates that if the current tools for the treatment and prevention of TB are not improved, nearly 1 billion additional people will become infected by *M. tuberculosis *between the 2000 and 2020. Among these, 200 million will develop the active disease and 35 million will die from TB. By the end of 2004, 199 (94%) of 211 countries notified 4.4 million new and relapsed cases, of which 1.9 million (44%) were new sputum smear-positive. Among these notifications, 3.7 million were from DOTS (Directly Observed Treatment Short course) areas. The Region of the Americas notified 235,187 cases in 2004 [1]. Venezuela covers an area of 916,495 km^2 ^and has a population of around 25 million inhabitants, and has shown a marked decrease in the incidence of TB during the last years. The TB prevalence rate in 1939 was 98.6 per 100,000 inhabitants for mortality and 111 per 100,000 inhabitants for morbidity [2]. Since then, the incidence has decreased dramatically to reach 6734 cases (26 per 100,000 inhabitants), with most being confined within the 15 to 45 year age group. Nevertheless, in indigenous populations, TB incidence has remained very high (137.4 per 100,000 inhabitants) [3]. In 1982, the National Tuberculosis Control Program of Venezuela (NTP) implemented the directly observed treatment and since 1994 has used a standardised six-month treatment for all new TB cases with or without culture confirmation [4].

Venezuela carried out the first survey of anti-tuberculosis drug resistance during 1998–1999 as part of the Global Project of Anti-tuberculosis Drug Resistance Surveillance, coordinated by the International Union Against Tuberculosis and Lung Disease (IUATLD) and the WHO. The survey enrolled 873 patients with smear-positive TB, and living in 23 states from Venezuela [4,5]

The molecular characterisation of the isolates collected during this study was not carried out and there is no information on the major circulating clades of *M. tuberculosis*. The mutations involved in drug resistance and the question of whether the Beijing family of the *M. tuberculosis *complex had spread into Venezuela have not been studied until now. Molecular epidemiological studies have showed the widespread of this family and its association with drug-resistance [6-8].

In recent years, the genetic typing of *M. tuberculosis *complex (MTC) strains has been widely used to support conventional epidemiological investigations of TB outbreaks and as a tool for studying transmission dynamics. The analysis of restriction fragment length polymorphisms (RFLP), which is based on the variability of the chromosomal location and copy number of the insertion sequence IS*6110*, is considered the gold standard for the genotyping of MTC [9,10]. However, this technique is expensive for developing countries, is cumbersome and requires a large amount of highly purified DNA, and is limited for strains with fewer than six IS*6110 *insertions or when rapid results are needed. Spoligotyping has emerged as a robust, easy, cheap and reliable supplementary tool for the molecular epidemiological study of TB. The establishment of an international database of spoligotypes provides information on the overall diversity of strain patterns. The combination of spoligotyping with bioinformatics has been proposed as a potential tool for defining major circulating clades of tuberculosis bacilli and to analyse the complexity of global TB transmission [11-14]. Spoligotyping has also been used to track TB epidemics, detect new outbreaks, define high-risk populations and to help to verify suspected false-positive cultures due to laboratory contamination and to confirm nosocomial transmission. In conjunction with other genotyping assays, it has also been used to investigate the evolutionary genetics of the tubercle bacilli [15-18].

This present investigation aims to provide an initial idea of the genetic biodiversity of *M. tuberculosis *in Venezuela. We used spoligotyping as a first-line discriminatory test in 670 strains isolated from the 23 surveyed states during the first anti-tuberculosis drug-resistance survey. RFLP analysis of IS*6110 *and characterisation of the *katG *and *rpoB *genes were carried out on 14 of the 18 MDR strains included in this survey.

## Results

### Study of population

A total of 873 patients with sputum smear-positive pulmonary TB were included in the survey to determine the initial prevalence and acquired resistance to the principal anti-TB drugs. Of these, 769 (88%) had never been treated for tuberculosis and the other 104 (12%) had previously received treatment (Table 1). Of the 873 isolates, 783 (90%) were sensitive to all four evaluated drugs and 90 (10.3%) showed resistance to at least one drug. Among the 769 cultures of the never treated patients, 58 (7,5%) were resistant to one or more drugs whereas the remaining 711 (92.5%) were susceptible to all four tested drugs. The rate of MDR was 0.5% (4) and 13.5% (14) in strains of never treated and previously treated patients respectively. Total resistance to INH and RIF in never treated patients was 4% (30 patients) and 1% (8 patients), respectively. This rate was 23% (24 patients) and 18.3% (19 patients) respectively in previously treated patients (Table 1). Most of the patients with drug-resistant isolates (74/90-82%) were aged between 15 and 54 years. Among the 90 patients resistant to one or more drugs, 72% were male.

### Analysis of the Spoligotyping patterns and comparison with an international database

**Figure 1:**
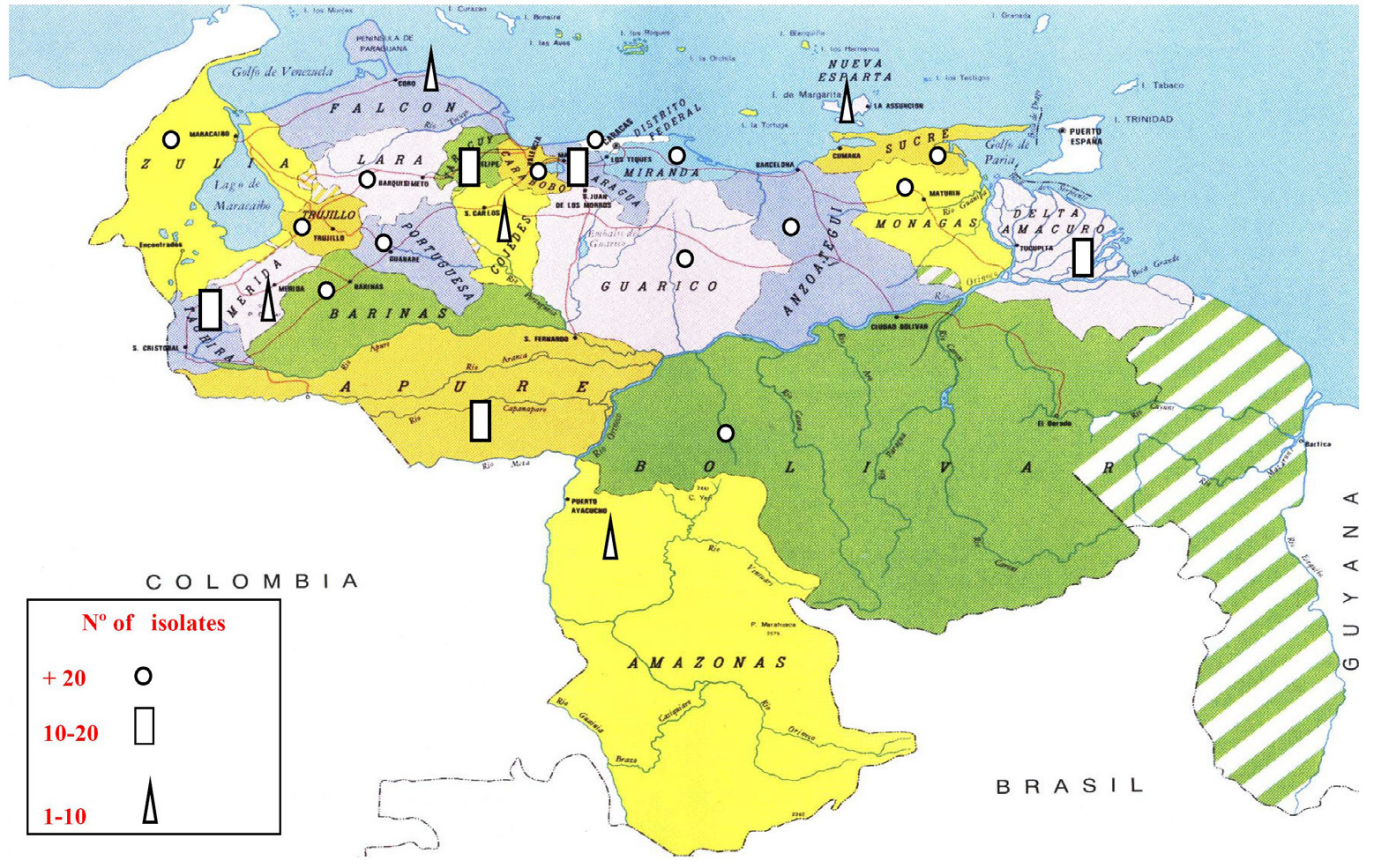
Distribution and number of isolates studied by spoligotyping in 23 provinces from Venezuela.

Of the 873 *M. tuberculosis *isolates from the national survey, 670 (77%) could be typed satisfactorily by spoligotyping. There were isolates of the 23 states surveyed among the genotyped strains (Fig 1). The spoligotype (SIT = Spoligo-International Type Number, a common pattern shared by two or more isolates) was determined by comparing our obtained patterns with those in the international spoligotype database of the Institute Pasteur of Guadeloupe [19]. At the time of the study, the database contained patterns for 29,363 clinical isolates, grouped into 1689 SITs. Of the 670 strains analysed, 79 (12%) were non-SIT (orphan patterns) according to spoligotype database and the remaining 591 (88%) isolates clustered into 102 SITs. Of these, 74 were found in the international database. The inclusion of our data to SpolDB4 created 28 new SITs, 20 of them were specifically created with Venezuelan isolates. These were identified with the SITs: 1691, 1692, 1693, 1696, 1698, 1699, 1700, 1701, 1702, 1703, 1704, 1705, 1707, 1711, 1713, 1714, 1715, 1716,1718 and 1719) (Table 2). The new SIT 1691 (characterised by the absence of spacers 3,13,15,21–24 and 33–36) comprised 14 strains isolated of nine provinces from Venezuela (Dtto. Federal, Carabobo, Aragua, Anzoátegui, Sucre, Apure, Barinas, Portuguesa and Bolivar). Whereas the remaining new endemic SITs comprised between two and five isolates. Eight new inter-region clusters were created (SITs: 1694, 1695, 1697, 1706, 1708, 1709, 1710, 1717) with one Venezuelan and one non-Venezuelan isolate from USA, France, Latvia, The Netherlands, Brazil and Australia (Table 2). Three isolates of indigenous patients from the Warao ethnic group (Delta Amacuro State) created the new clusters SITs 1716, 1718 and the inter-regional SIT 1717.

Of the 591 isolates, 548 (93%) were grouped into 59 clusters (the number of strains in each cluster ranged from 2 to 112). Among the 59 clusters, 18 contained five or more isolates, which we defined as major spoligotypes. The total number of these isolates comprised 80% (n = 443) of the clustered isolates. The major SITs were: 17, 42, 93, 53, 20, 605, 1691, 60, 905, 118, 50, 51, 333, 960, 34, 194, 1696 and 47 (Table 2).

The SITs found in our study allowed the classification of the isolates into representative patterns describing families or clades [14,20], as 74% (411/556) belong to the Latin American and Mediterranean (LAM) family (sub-clades 2,9,5,1,4,3,6), 13% (70/556) belong to the ill-defined T (sub-clades 1,2,3,4,5), 3% (16/556) belong to the Haarlem family (sub-clades 1 and 3), 1%(1/556) grouped to the S family, and 9% (51/556) were classified undesignated (SITs: 605, 905, 960, 397, 1692, 162, 1698, 106, 239, 443, 606 and 821) (Table 2). The geographic distribution (C1) [20] of the shared types was as follows: 24 SITs were ubiquitous types (Ubiq) or are widespread in the world, 20 SITs were endemic (Venezuela) and 23 SITs were geographically limited (Loc) (Table 2). We observed no strains belonging to the Beijing family among the 670 clinical isolates analysed.

### Phylogeny reconstruction

**Figure 2:**
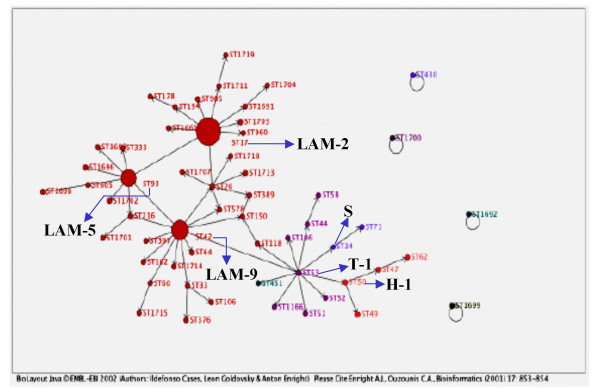
**Genetic network of spoligotypes detected in Venezuela using parsimony principles and the Biolayout software**. Each black arrow represents a phylogenetic link between two spoligotypes differing by a genetic change. The size of the circles representing the spoligotypes is proportional to the number of isolates in each cluster. Prevalent phylogenetic clades in Venezuela: LAM (2-9-5); T-1; Haarlem (H-1) and S are named. See text for details.

We used Biolayout software to build a genetic network of the SITs detected in Venezuela (Fig. 2) [22]. In this model, the size of the circles indicating the SIT is proportional to their quantitative distribution in this study. We used maximum parsimony principles and assumed an underlying evolution model of the DR locus by deletion of single or contiguous direct variable repeats (DVRs) to link most, if not all, of the spoligotypes found in this study. Each arrow represents a phylogenetic link between two spoligotypes that differ by a genetic change, whether this change corresponds to single or many contiguous DVRs deletion. Fig 2 shows the phylogenetic clades prevalents as well as that three variants of the LAM family – LAM2 (SIT17), LAM9 (SIT42), and LAM5 (SIT93) – constitute the backbone of the genetic TB population-structure in Venezuela. A more detailed picture will require further investigations using complementary genetic markers.

### Analysis by IS6110-RFLP typing, *katG *and *rpoB *genes characterisation

**Figure 3:**
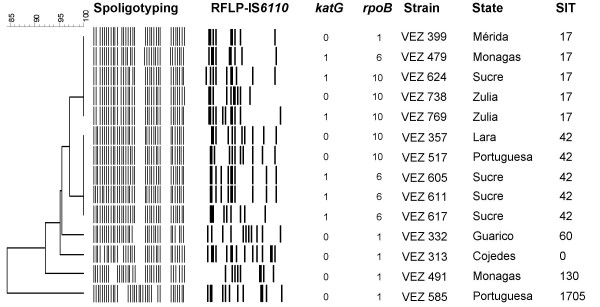
**IS*6110 *RFLP, *katG *and *rpoB *gene mutations of 14 MDR isolates clustered by Spoligotyping**. Dendogram based on the Spoligotyping method of 14 MDR strains showing the IS*6110 *RFLP, the codon 315 in *katG *gene (0: No Ser315Thr mutation, 1: Ser315Thr) and *rpoB *gene mutations (1: No mutation, 6: Asp516Val/GAC-GTC, 10: Ser531Leu/TCG-TTG). Strain: identification of the isolate. State: origin of the isolate. SIT: spoligo-international type number [19].

We analysed 14 (78%) of the 18 isolates identified as MDR using phenotypic methods. While Spoligotyping grouped 10 strains in 2 clusters (SITs 17 and 42) and 4 unique isolates were identified as SIT 60, 130, 1705 and 0 (orphan pattern), RFLP analysis classified eight (57%) isolates as unique, with the other six (43%) isolates being grouped into three clusters of two isolates (Fig 3). The number of IS*6110 *per strain varied from 9 to 15. Of the three clusters, two contained isolates from patients from two different states and one cluster contained isolates from patients living in the same state (Fig 3). We investigated these isolates to identify the mutation associated with INH resistance (*katG *gene) and RIF resistance (*rpoB *gene). As summarized in the Fig 3, we found the Ser315Thr mutation in the *katG *gene in 6 (43%) of the 14 isolates, identified as: VEN 479, 624, 769, 605, 611 and 617.

Mutations in the *rpoB *gene were detected in 9 (64%) of the 14 isolates: VEN 479, 624, 738, 769, 357, 517, 605 611 and 617. No *rpoB *mutations were found in 5 (36%) of the 14 strains phenotypically identified as MDR, which corresponded to 1 previously treated patient and 4 never treated patients. Two different mutations were observed, which occurred in codon 531 and codon 516 (Fig 3). The specific mutation TCG-TTG (Ser/Leu) of the codon 531 was detected in 5 isolates (VEN 624, 738, 769, 357, 517) and the mutation GAC-GTC (Asp/Val) of the codon 516 was found in 4 isolates (VEN 479, 605, 611 and 617). Six (43%) of the 14 isolates had mutations in both the *katG *and *rpoB *genes (VEN 479, 624, 769, 605, 611 and 617).

Finally, when the three clusters were studied by RFLP analysis of IS*6110*, spoligotyping, and mutations of the *katG *and *rpoB *genes, we found that only two of them were identical by the four different methods, presumably revealing cases of recent transmission of MDR tuberculosis (Fig 3).

## Discussion

In 1994, the WHO and IUATLD launched a Global Project on Anti-Tuberculosis Drug resistance surveillance to assess the global extent of drug resistance. During 1998–1999, the NTP in Venezuela carried out the first national drug resistance survey through out the entire country for a period of nine months. The sampling method was the proportionate cluster method and the total of 873 patients were tested. The Venezuelan NTP has a long history of tuberculosis control and the results of the survey showed a very low prevalence (0.5%) of MDR-TB in never treated patients, similar to those reported in Canada, Cuba, Uruguay, and Chile, which are countries with effective NTCPs [4,5]. However, the prevalence of resistance to at least one drug among the previously treated cases was 30.8% and MDR-TB was 13.5%.

We used spoligotyping to molecularly characterise 670 (77%) of the 873 isolates, including isolates of the 23 states surveyed. It was not possible to analyse all isolates by RFLP analysis IS*6110*. Nevertheless, as the molecular epidemiology of MDR *M. tuberculosis *in Venezuela is not know, we investigated the most of MDR isolates detected in the survey using spoligotyping, RFLP analysis of IS*6110 *and point mutations of the *katG *and *rpoB *genes.

Comparison of our spoligo-patterns with the international spoligotyping database revealed that 74 SITs had already been identified. We describe 20 new SITs specific to Venezuela that were found in 75% of the 23 provinces surveyed and show that these types are extensively dispersed. The six most prevalent spoligotypes (SITs 17, 42, 93, 53, 20 and 605) included the half of all the isolates. Among the clades of *M. tuberculosis *complex described as harbouring specific spoligotypes, 89% of the isolates studied belonged to three major genotypic families (LAM, T and Haarlem). The majority of them belonged primarily to the LAM family, which has a high prevalence in Latin-America, the Caribbean and the Mediterranean region. This family recently was reclassified in 12 sub-clades [21] and in this study we identified SITs corresponding to the sub-clades LAM-2, 9, 5, 1, 4, 3 and 6. However, the genetic network of spoligotypes showed that the LAM-2, LAM-9, and LAM-5 do indeed constitute the backbone of the genetic TB population-structure in Venezuela. A more detailed picture of this network would require further investigations using complementary genetic markers to better identity the clinical isolates within the LAM superfamily of *M. tuberculosis*, which is highly prevalent in Venezuela.

The "ill-defined" T genetic family encountered in different regions of the world [20] was the second most common family. Within this family we found SITs that belonged to the sub-clades T-1, 2, 3, 4 and 5 [21]. We also found some isolates belonging to the Haarlem family, sub-clades H-1 and H-3. This family has a European origin and has been described in the Caribbean and Central America, suggesting that it is remnant of the European colonisation [23,24]. The Beijing/W genotype was not detected in this study.

Our finding of seven new isolates belonging to SIT 333 (LAM-5), which has been reported in Guadeloupe, French Guiana, Haiti and Venezuela, suggest a common origin. SIT 605 had been reported in the international database as only being found in Venezuela and the USA, and our study adds other 18 clinical isolates, to this SIT, from patients living in seven Venezuelan provinces. Further studies are needed for a better understanding of the distribution of these spoligopatterns.

When the Venezuelan isolate spoligopatterns were added to the international database, eight new inter-regional clusters were created by matches with identical patterns from USA, Brazil, France, Latvia, Australia and The Netherlands. Curiously, one of these new inter-regional clusters (ST 1717), which included an Australian isolate, shared a pattern with one of our Venezuelan isolates belonging to an indigenous patient from the Delta Amacuro state.

We characterised the *katG *and *rpoB *genes in 78% (14/18) of the MDR strains isolated in the survey. We observed that 64% of the isolates had a mutation in the *rpoB *gene, whereas other studies have reported mutations in the 81-bp core region in more 90% of MDR strains [25-27]. It has been reported that a mutation outside of the 81-bp *rpoB *region associated with RIF resistance, and the heteroresistance caused by a mixture of mycobacterial subpopulations with different susceptibilities to RIF may influence the sensitivity of the molecular tests for detecting resistance [28]. In our case, the rifampicin-resistance of these strains had been confirmed by two phenotypic methods, however the mutation in the *rpoB *gene was no detected in five of the fourteen strains analysed. Nevertheless, a new test to confirm the resistance of those strains could not be carried out because the cultures of the strain genotyped were not available. The Ser531Leu and Asp516Val mutations detected in this study are among the most common observed worldwide [29-31].

Mutations in the *katG *gene have been reported between 20 and 80% of INH resistant *M. tuberculosis *strains. The mutation Ser-Thr at codon 315 of *katG *is the most common [32]. In our study, 43% of the isolates showed this mutation, consistent with other studies [33,34]. Other mutations in of this gene and other gene regions, such as the *inhA *locus, the *kasA*, *ndh, oxyR-ahpC *intergenic region, have been implicated in INH resistance. However, we did not evaluate these mutations.

The characterisation of *rpoB *could discriminate between the three clusters grouped by RFLP analysis of IS*6110 *and by spoligotyping, as only one showed identical mutations suggesting a recent transmission of MDR tuberculosis [31]. This cluster contained isolates recovered from the same province. The other two clusters had different resistance-associated mutations, indicating that these isolates had acquired mutations independently, and were probably transmitted before the acquisition of drug resistance.

There is limited information regarding the distribution of *M. tuberculosis *complex genotypes as well as few molecular studies on MDR strains have been carried out in the most Latin American countries. A recent study describing the genetic diversity of *M. tuberculosis *complex and the world-wide distribution of spoligopatterns [21] showed only a limited number of registered strains, particularly in regions outside the immediate vicinity of Venezuela. Greater financial means are needed to implement an adequate infrastructure and to develop molecular typing techniques for the national reference laboratory at NTP, which will be an important tool for the control programmes in these countries.

Although Venezuela has had, a well functioning NTP during many years, the proportion of immigrants from neighbouring countries with a high incidence of TB and MDR strains, the reduction of TB control activities, and a similar decrease in the locating and treatment of cases among indigenous populations may worsen the current TB situation in the coming years. Other national studies incorporating other molecular epidemiology methods are needed to evaluate the actual situation of MDR in Venezuela.

## Conclusion

As it is important to clarify the molecular epidemiological characteristics for effective tuberculosis control, we used spoligotyping as a first-line discriminatory test. This is the first genetic biodiversity study of *M. tuberculosis *in Venezuela and our results should be considered a starting point for the creation of a national genotype database to support the National Tuberculosis Control Program in Venezuela.

## Methods

### Bacterial strains, drug resistance assays and patients

The study used isolates collected within the framework of first anti-tuberculosis drug-resistance survey carried out during 1998–1999 by the NTP of Venezuela. This study included patients living in the 23 provinces of Venezuela, with no distinction of sex and age. Information on the patients – sex, age, and previous history of TB and treatments – were collected by a standard questionnaire for all patients presenting with bacteriologically confirmed TB by the laboratories of the NTP.

The specimens from patients were collected and sent to the National Reference Laboratory (NRL) at the NTP for culture in Lowenstein-Jensen medium. All isolates were identified as *M. tuberculosis *complex using biochemical tests: production of niacina, catalasa activity, nitrate reduction, pigment production and growth. Drug susceptibility testing against Isoniazid (INH), Rifampicin (RIF), Ethambutol (ETB) and Streptomycin (STM) was carried out using the proportion method [35] and the BACTEC 460 radiometric method [36]. The supranational laboratory assigned by the Pan American Health Organization/WHO to Venezuela was the National Institute for Food Protection and Zoonosis (INPAZ), Argentina, which was later replaced by the National Laboratory of Santiago, Chile, as the supranational laboratory. Spoligotyping of 670 (77%) of the 873 clinical isolates, was carried out in the Laboratory of Mycobacterial Genetics, University of Zaragoza, Spain. The remaining isolates could not be analysed because some presented or contaminated cultures what prevented the analysis or the poor quality of the DNA showed not clearly defined spoligopatterns. A sufficient quality of genomic DNA for IS*6110 *RFLP fingerprinting was obtained from 14 of the 18 MDR strains isolated in the survey.

### Molecular typing

#### Spoligotyping

Spoligotyping was carried out as previously described [11]. The DR region was amplified with the oligonucleotides:DRa: 5'-ggttttgggtctgacgac-3'(biotinylated 3') – DRb: 5'ccgagaggggacggaaac-3'. The amplified biotinylated products were hybridised to a set of 43 oligonucleotides covalently bound to the spoligo-membrane (Isogen Biosciences B.V., Marseen, The Netherlands). Each of these oligonucleotides represented a known spacer sequence within the DR locus. DNAs from *M. tuberculosis *type isolate H37Rv and *M.bovis *BCG, as well as sterile water, were used as controls. The hybridised PCR products were incubated with streptavidin-peroxidase conjugate and the membrane was then exposed to the chemiluminescence system (Amersham, Little Chalfont, England) followed by exposure to X-ray film (Hyperfilm ECL, Amersham), according to the manufacturer's instructions. The X-ray film was developed using standard photochemical procedures after two hours of exposure. The films were scanned and analysed using Bionumerics program, version 4.0 (Applied Maths, Kourtrijk, Belgium).

#### RFLP analysis of IS*6110*

RFLP analysis was carried out using Southern blot transfer and DNA hybridisation with IS*6110 *according to the internationally standarised methodology [9]. Briefly, chromosomal DNA extracted by the CTAB method and digested with *Pvu*II was separated by electrophoresis in an agarose gel. After electrophoresis, restriction fragments were blotted onto nylon membrane using a vacuum blotter and hybridised with a peroxidase-labelled 867-bp IS*6110*-specific sequence. *M. tuberculosis *strain 14323 was used as a marker to allow comparison of the IS*6110 *patterns. The ECL Direct Labelling and Detection System (Amersham Biosciencies) was used for probe labelling and detection of hybridisation signals. The films (Hyperfilm ECL; Amersham) were scanned and the patterns compared.

#### Molecular characterisation of drug resistance

A point mutation in codon 315 of the catalase-peroxidase gene (*katG*) linked to INH-resistance were detected as described by Uhl *et al*. [37], and any mutations within an 81-bp fragment of the *rpoB *gene for RIF resistance were investigated. Between 10 and 100 ng of DNA was added to a reaction tube containing a PCR mixture: 50 mM KCl, 10 mM Tris-HCl, 1.5 mM MgCl_2_, 200 μM [each] deoxynucleoside triphosphate, 2.5 U of Taq polymerase (pure Re Taq™ Ready-To Go™ [PCR] Beads- Amersham, Biosciences), sterile deionised water and 2.5 μM of each primer.

A 620-bp portion of the *katG *gene was amplified with primers KatG 904: 5'-agctcgtatggcaccggaac-3' and KatG 523: 5'-ttgacctcccacccgacttg-3', and the resulting product was digested with *Msp*I (Boehring Mannheim). After separation in a 3% agarose gel, the fragments were visualised by ethidium bromide staining and exposure to UV light.

The *rpoB *gene was amplified with primers rpoB1: 5'-tacggtcggcgagctgatcc-3' and rpoB2: 5'-tacggcgtttcgatgaacc, yielding a 432 bp fragment containing the hot-spot region. PCR products were purified with a commercial kit (ExoSAP-IT-USB Corporation). After sequencing (Servicio de Secuenciación de DNA-CNIO. Madrid, Spain) BLASTn software was used for DNA sequence comparisons [38].

#### Computer analysis

The patterns obtained by the different methods were compared. The dendrograms were constructed using the unweighted-pair group method using average linkages (UPGMA) after pairwise comparison of strains by calculating the Dice coefficient. Optimisation was set at 1% and position tolerance at 1.5%.

The spoligotyping results were entered in a binary format (1 and 0) according to the hybridisation results (positive or negative, respectively) as Excel (Microsoft, CA) spreadsheets and compared to the updated international spoligotyping database of the Pasteur Institute of Guadeloupe [19]. Only patterns with 100% similarity were considered as clusters. At the time of the study, the updated SpolDB4 contained 29,363 patterns distributed into 1689 SITs and 2,860 orphan patterns consisting of entries occurring only once in the database (SIT is defined as an identical spoligotype in two or more isolates). Some of the patterns were included in the clades or families already described [14,20]. A cluster of *M. tuberculosis *was defined as two or more isolates with identical spoligotyping or IS*6110 *RFLP patterns.

### Phylogeny reconstruction

Biolayout software using parsimony principles [22] was used to construct a genetic network of spoligotypes found in Venezuela. In the model shown in Figure 2, each arrow represents a likely evolutionary link between two spoligotypes differing by a single genetic change, whether by loss of a single or of many direct variable repeats (DVRs), and the size of the circles indicating the spoligotypes is proportional to the number of isolates in the cluster.

## Authors' contributions

LA conceived the study, carried out the molecular genetic studies, analysed the data and drafted the manuscript; RA, ME, AG and AVdeS participated in the design and carried out the first survey of anti-tuberculosis drug-resistance, analysed the data, and provided the clinical isolates for molecular study. CR and JR carried out mycobacteriological diagnostics, isolation of clinical isolates, identification, drug susceptibility tests and provided information about the clinical isolates; CL participated in the genotyping studies; AC assisted with data entry, conducted the bioinformatics programming and statistical analysis; MAL and MJR provided technical help in the conservation of the strains; CS and NR carried out the phylogeny reconstruction studies, participated in the identification and designation of the SITs and also helped draft the manuscript; CM participated in the design of the study, and provided critical comments for the manuscript; SS conceived the study, and participated in its design, coordination of the investigation, and helped to draft the manuscript for consideration for publication. All authors contributed to the study, read and approved the final manuscript.

## Figures and Tables

**Table 1:** Prevalence of *M. tuberculosis *drug resistance in Venezuela, 1998–1999.

	**Never treated** n(%)	**Previously treated cases** n(%)	**Total** n(%)
Total number of cultures	769 (88)	104 (12)	873(100)
Susceptible to all drugs	711(92.5)	72 (69.2)	783(90)
Resistant to one or more drugs	58 (7.5)	32(30.8)	90(10.3)
Resistant to only one drug	38(4.9)	12(1.5)	50(5.7)
H	13(1.7)	6(5.8)	19(2.1)
R	3(0.4)	3(2.9)	6(0.6)
E	1(0.1)	0(0.0)	1(0.1)
S	21(2.7)	3(2.9)	24(2.7)
MDR (Multidrug resistance)	4(0.5)	14(13.5)	18(2.1)
H+R	2(0.3)	4(3.8)	6(0.7)
H+R+S	1(0.1)	5(4.8)	6(0.6)
H+R+E	0(0.0)	2(1.9)	2(0.2)
H+R+E+S	1(0.1)	3(2.9)	4(0.4)
Other patherns	16(2.1)	6(5.8)	22(2.5)
H+E	2(0.3)	1(1.0)	3(0.3)
H+S	10(1.3)	3(2.9)	13(1.5)
E+S	2(0.3)	0(0.0)	2(0.2)
R+E	1(0.1)	0(0.0)	1(0.1)
H+E+S	1(0.1)	0(0.0)	1(0.1)
R+E+S	0(0.0)	2(1.9)	2(0.2)

Never treated patients

Total H resistance: 30/769 × 100 = 4%

Total R resistance: 8/769 × 100 = 1%

Total E resistance: 8/769 × 100 = 0.9%

Total S resistance: 36/769 × 100 = 0.7%

Total MDR resistance: 4/769 × 100 = 0.5%

Previously treated patients

Total H resistance: 24/104 = 23%

Total R resistance: 19/104 = 18.3%

Total E resistance: 8/104 × 100 = 7.7%

Total S resistance: 16/104 × 100 = 15.3%

Total MDR resistance: 14/104 = 13.5%

Results of the first survey of anti-tuberculosis drug resistance in Venezuela during 1998–1999.

Resistance to drugs: Isoniazid (H), Rifampicin (R), Ethambutol (E), Streptomycin (S) in never treated and previously treated patients.

**Table 2:** Description of the *M. tuberculosis *clusters found in Venezuela.

^1^**SIT**	**Spoligotyping patterns -Binary description**	^2^**n**	**%**	^3^**Clade**	^4^**C-1**
17		122	21.94	LAM-2	Ubiquitous
42		66	15.47	LAM-9	Ubiquitous
93		70	12.59	LAM-5	Ubiquitous
53		26	4.6S	T-1	Ubiquitous
20		26	4.63	LAM-1	Ubiquitous
605		18	3.24	U	Loc (VEN/USA)
1691		14	2.52	LAM-2	Endemic (VEN)
60		13	2.39	LAM-4	Ubiquitous
905		10	1.60	U	Loc (VEN-USA-ARG)
118		9	1.61	T-2	Ubiquitous
50		9	1.61	H-3	Ubiquitous
51		8	1.43	T-1	Ubiquitous
333		7	1.25	LAM-5	Loc (GUP-GUF-HTI-VEN)
960		5	0.90	U	Loc (VEN-USA)
34		5	0.90	S	Ubiquitous
194		5	0.90	LAM-4	Loc (USA-BRA-MEX-VEN)
1696		5	0.90	LAM-5	Endemic (VEN)
47		5	0.90	H-1	Ubiquitous
130		4	0.71	LAM-3	Ubiquitous
150		4	0.71	LAM-9	Ubiquitous
397		4	0.71	U	Loc (ITA-BRA-ARG-USA-VEN)
1692		4	0.71	U	Endemic (VEN)
1700		4	0.71	T-1	Endemic (VEN)
33		3	0.54	LAM-3	Ubiquitous
44		3	0.54	T-4	Ubiquitous
58		3	0.54	T-5	Ubiquitous
64		3	0.54	LAM-6	Ubiquitous
162		3	0.54	U	Loc (ARG-VEN-USA-FIN-FXX-ITA)
376		3	0.54	LAM-3	Loc (VEN-USA)
578		3	0.54	LAM-9	Loc (USA-VEN)
1698		3	0.54	U	Endemic (VEN)
1701		3	0.54	LAM-9	Endemic (VEN)
1702		3	0.54	LAM-5	Endemic (VEN)
1705		3	0.54	T-1	Endemic (VEN)
1713		3	0.54	LAM-1	Endemic (VEN)
1718		3	0.54	LAM-9	Endemic (VEN)
52		2	0.36	T-2	Ubiquitous
62		2	0.36	H-1	Ubiquitous
71		2	0.36	S	Ubiquitous
106		2	0.36	U	Loc (CUB-ECU-M EX-USA-VEN)
132		2	0.36	U	Loc (ESP-AUS-BRA-VEN)
167		2	0.36	T	Ubiquitous
178		2	0.36	LAM-4	Loc (CUB-SEN-VEN)
196		2	0.36	T-1	Ubiquitous
386		2	0.36	LAM-9	Ubiquitous
389		2	0.36	LAM-1	Loc (BRA-PRT-FIN-VEN)
430		2	0.36	T-4	Loc (USA-VEN)
1166		2	0.36	T-1	Ubiquitous (MWI-USA-VEN)
1661		2	0.36	LAM-2	Loc (VEN-BRA)
1693		2	0.36	LAM-5	Endemic (VEN)
1699		2	0.36	LAM-9	Endemic (VEN)
1703		2	0.36	LAM-9	Endemic (VEN)
1704		2	0.36	LAM-5	Endemic (VEN)
1707		2	0.36	T-1	Endemic (VEN)
1711		2	0.36	LAM-2	Endemic (VEN)
1714		2	0.36	LAM-9	Endemic (VEN)
1715		2	0.36	LAM-4	Endemic (VEN)
1716		2	0.36	T-3	Endemic (VEN)
1719		2	0.36	LAM-2	Endemic (VEN)
1694		1	0.18	LAM-5	Loc (VEN-USA)-Cluster inter-region
1695		1	0.18	LAM-2	Loc (VEN-BRA)-Cluster inter-region
1697		1	0.18	LAM-9	Loc (VEN-FXX)-Cluster inter-region
1706		1	0.18	LAM-9	Loc (VEN-USA)-Cluster inter-region
1708		1	0.18	LAM-9	Loc (VEN-LVA)-Cluster inter-region
1709		1	0.18	LAM-5	Loc (VEN-USA)-Cluster inter-region
1710		1	0.18	LAM-4	Loc (VEN-NLD)-CIuster inter-region
1717		1	0.18	X-1	Loc (VEN-AUS)-Cluster inter-region

Total		556	100		

^1^SIT, designation of the spoligotype in the international database [19]. ^2^n: Number of strains. ^3^Clade: defined mainly as described [20, 21] LAM: Latin American and Mediterranean, H: Haarlem, T (ill-defined T clade), S: S clade, X: X clade, U: Undesignated. ^4^C-1: qualifier that tentatively defines an SIT as Endemic, Loc: localized or ubiquitous, as defined in Filliol *et al*. [20]. Geographic distribution: VEN: Venezuela, USA: United States of America, BRA: Brazil, GUP: Guadeloupe, GUF: French Guiana, HTI: Haiti, ESP: Spain, ECU: Ecuador, ARG: Argentina, CUB: Cuba, MEX: Mexico, SEN: Senegal, PRT: Portugal, FIN: Finland, ITA: Italy, MWI: Malawi, FXX: Metropolitan France, LVA: Latvia, NLD: The Netherlands, AUS: Australia.
